# Single-Dose Toxicity of Individual and Combined Sterigmatocystin and 5-Methoxysterigmatocistin in Rat Lungs

**DOI:** 10.3390/toxins12110734

**Published:** 2020-11-23

**Authors:** Daniela Jakšić, Ida Ćurtović, Domagoj Kifer, Dubravka Rašić, Nevenka Kopjar, Vedran Micek, Maja Peraica, Maja Šegvić Klarić

**Affiliations:** 1 Faculty of Pharmacy and Biochemistry, University of Zagreb, 10000 Zagreb, Croatia; djaksic@pharma.unizg.hr (D.J.); icurtovic@student.pharma.unizg.hr (I.Ć.); dkifer@pharma.unizg.hr (D.K.); 2 Institute for Medical Research and Occupational Health, 10000 Zagreb, Croatia; rasic@imi.hr (D.R.); nkopjar@imi.hr (N.K.); vmicek@imi.hr (V.M.); mperaica@imi.hr (M.P.)

**Keywords:** Aspergilli series *Versicolores*, sterigmatocystin, 5-methoxysterigmatocystin, intratarcheal instillation, genotoxicity, pro-inflammatory cytokines

## Abstract

Sterigmatocystin (STC) and 5-methoxysterigmatocystin (5-M-STC) are mycotoxins produced by common damp indoor Aspergilli series *Versicolores*. Since both STC and 5-M-STC were found in the dust of indoor occupational and living areas, their occupants may be exposed to these mycotoxins, primarily by inhalation. Thus, STC and 5-M-STC were intratracheally instilled in male Wistar rats using doses (0.3 mg STC/kg of lung weight (l.w.); 3.6 mg 5-M-STC/kg l.w.; toxin combination 0.3 + 3.6 mg/kg l.w.) that corresponded to concentrations detected in the dust of damp indoor areas in order to explore cytotoxicity, vascular permeability, immunomodulation and genotoxicity. Single mycotoxins and their combinations insignificantly altered lactate-dehydrogenase activity, albumin, interleukin-6, tumor necrosis factor-α and chemokine macrophage inflammatory protein-1α concentrations, as measured by ELISA in bronchioalveolar lavage fluid upon 24 h of treatment. In an alkaline comet assay, both mycotoxins provoked a similar intensity of DNA damage in rat lungs, while in a neutral comet assay, only 5-M-STC evoked significant DNA damage. Hence, naturally occurring concentrations of individual STC may induce DNA damage in rat lungs, in which single DNA strand breaks prevail, while 5-M-STC was more responsible for double-strand breaks. In both versions of the comet assay treatment with STC + 5-M-STC, less DNA damage intensity occurred compared to single mycotoxin treatment, suggesting an antagonistic genotoxic action.

## 1. Introduction

Sterigmatocystin (STC) is one of the most commonly occurring polyketide mycotoxins in damp occupational and indoor living areas [1,2,3], principally produced by *Aspergillus* section *Nidulantes* series *Versicolores* that can be found in indoor damp occupational and living environments [4]. Recent studies have shown that among *Aspergilli* series *Versicolores*, the most frequent contributors of STC in dust of occupational and/or residential environments were the species *A. jensenii* and *A. creber*, followed by *A. protuberus*, *A. puulaauensis*, *A. tennesseensis*, *A. venenatus*, *A. amoenus, A. griseoaurantiacus*, *A. fructus* and *A. pepii* [5,6,7]. 5-Methoxysterigmatocystin (5-M-STC) is produced in association with STC by some Aspergilli series *Versicolores*, sometimes at higher levels than STC [8]. 

STC is a precursor in aflatoxin biosynthesis and is therefore structurally related to aflatoxins [9]. Similarly to aflatoxin, STC is activated by the liver cytochrome P450 (CYP450) system with reactive epoxide that forms DNA adducts with guanine, and this is considered the underlying mechanism of STC genotoxicity [9]. STC-induced tumors, including hepatocellular carcinomas, hemangiosarcomas of the liver and pulmonary adenomas, resulted in its classification as a 2B carcinogen (possible human carcinogen) by the International Agency for Research on Cancer (IARC) [9,10]. STC induces lung adenocarcinoma in mice, genotoxicity and G_2_ cell cycle arrest in human immortalized bronchial epithelial BEAS-2B cells, human lung adenocarcinoma A549 cells and human esophageal epithelial Het-1A cells, and it is more cytotoxic than aflatoxin to A549 cells [11,12,13]. Intratracheally instilled STC in white Swiss Webster mice modulated inflammation-associated genes after 4 h of treatment, while after 12 h of instillation, mucus production and inflammation of the bronchiolar and alveolar epithelium and alveolar edema appeared [14]. 

The toxic properties of 5-M-STC have been poorly investigated to date. In terms of toxicity, a TA100 *Salmonella typhimurium* mutagenicity assay showed that 5-M-STC is mutagenic in the presence of metabolic activation [15] and is cytotoxic and genotoxic to A549 cells [16,17]. Both STC and 5-M-STC are detoxified through conjugation in primary tracheal epithelial cells. While STC is activated by CYP enzymes producing reactive epoxide, no such metabolite was detected with 5-M-STC [8,18].

In our recent study [6] in damp dwellings, 75 fungal metabolites were detected in dust samples and STC and 5-M-STC were among the dominant mycotoxins. The highest concentration of STC was 0.59 µg/g, while 5-MET-STC was recovered at a maximum level of 7.70 µg/g. Considering the reported frequent occurrence of STC- and 5-M-STC-producing *Aspergilli* series *Versicolores* [2,7,19], these two mycotoxins could be frequently expected in indoor damp occupational and living environments. Taking into account the maximal concentrations of STC (0.59 µg/g) and 5-M-STC (7.70 µg/g) found in dust, exploring the cytotoxic, inflammatory and genotoxic effects of STC and 5-M-STC alone, as well as their combination, in the lungs of male Wistar rats upon a single intratracheal instillation of mycotoxins was justified. Concentrations used for animal treatment were calculated according to following data related to human respiratory exposure to particulate matter (PM_2.5_): (i) average daily inhalation of adults is 10–12 m^3^ and the average concentration of PM_2.5_ could be up to 54 μg/m^3^ [20,21]; (ii) by multiplying these values, we can assume that the average daily inhaled PM_2.5_ could be up to 648 µg; (iii) thus, the daily inhaled STC and 5-M-STC would be 0.4 µg and 5 µg, respectively. These levels of STC and 5-M-STC were used in the experiment.

## 2. Results

Bronchioalveolar lavage fluid (BALF) was used as a sample for measuring (ELISA) lactate dehydrogenase (LDH) activity as an indicator of cytotoxicity, albumin as an indicator of vascular permeability and the cytokines interleukin-6 (IL-6), tumor necrosis factor-α (TNF-α) and chemokine macrophage inflammatory protein-1 (MIP-1α) as indicators of inflammation. Homogenized rat lungs were used as samples for measuring the levels of DNA damage by alkaline and neutral comet assays.

### 2.1. LDH Activity

**Figure 1:**
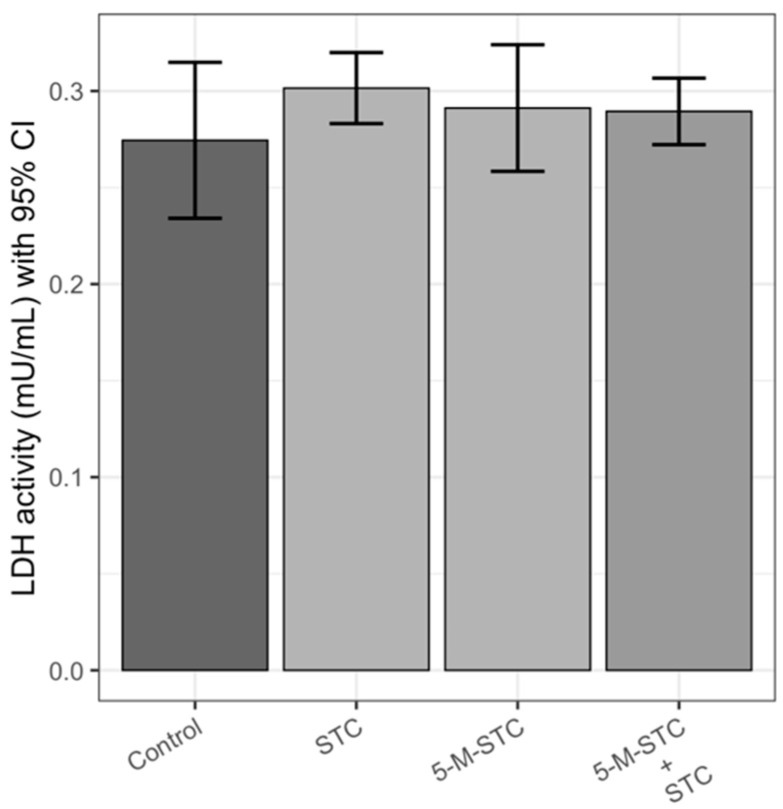
Lactate dehydrogenase (LDH) activity in bronchioalveolar lavage fluid (BALF) of Wistar rats upon 24 h of intratracheal instillation of mycotoxins. Each experimental group comprised six animals. Bar height reflects mean LDH activity of each group in nmol/mL/min (mU/mL), error bars present upper and lower limit of 95% confidence interval (CI). Abbreviations: STC—sterigmatocystin, 5-M-STC—5-metoxysterigmatocysin. Control rats were treated with 0.3% DMSO.

Cytotoxicity expressed as LDH activity levels measured in BALF upon 24 h of intratracheal instillation of mycotoxins is presented in Figure 1. STC and 5-M-STC alone and their combinations increased LDH activity in treated rats at similar levels, but without significant differences compared to control rats (*p* > 0.05).

### 2.2. Albumin

**Figure 2:**
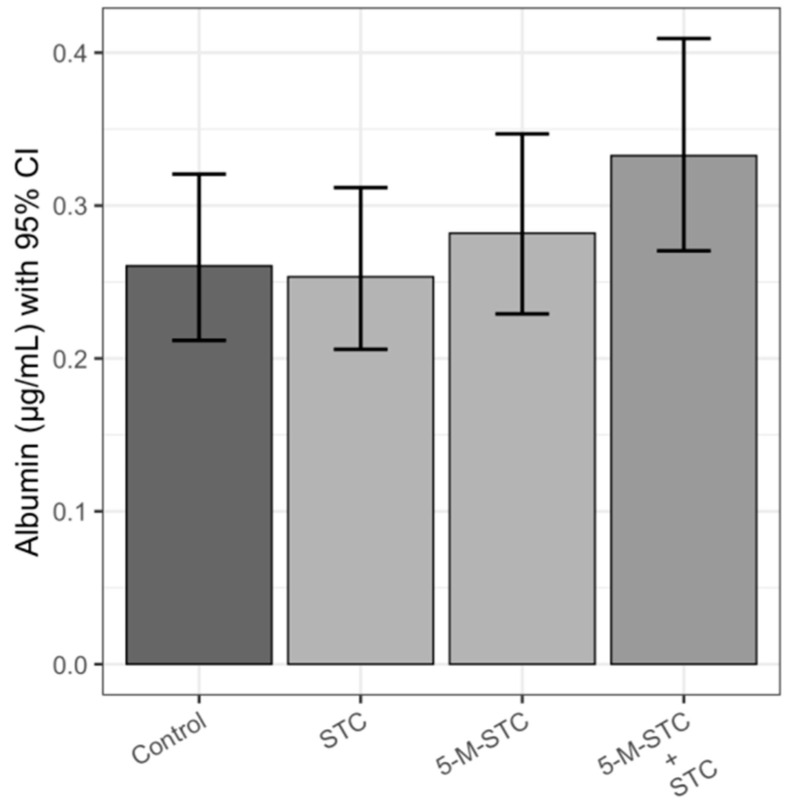
Albumin concentrations in BALF of Wistar rats upon 24 h of intratracheal instillation of mycotoxins. Each experimental group comprised six animals. Bar height reflects mean albumin concentration of each group in μg/mL, error bars present upper and lower limit of 95% confidence interval (CI). Abbreviations: STC—sterigmatocystin, 5-M-STC—5-metoxysterigmatocysin. Control rats were treated with 0.3% DMSO.

Albumin concentration in BALF was measured as a nonspecific indicator of vascular permeability. The increase in albumin concentration was detected in rats instilled with 5-M-STC and the combination STC + 5-M-STC, but these changes were not associated with a significant difference with respect to albumin concentration in control animals (*p* > 0.05) (Figure 2).

### 2.3. Pro-Inflammatory Cytokines and Chemokine

**Figure 3:**
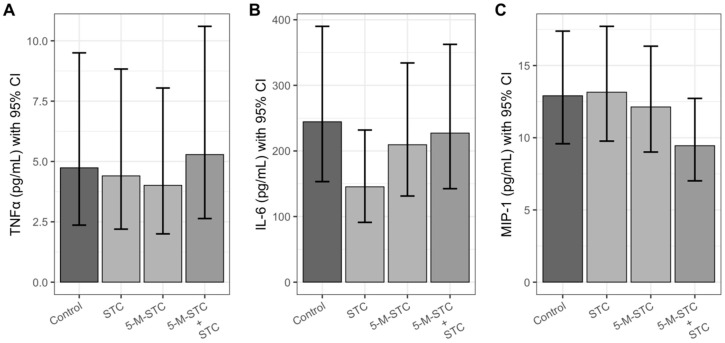
Cytokines (TNF-α and IL-6) and chemokine (MIP-1α) concentration in BALF of Wistar rats upon 24 h of intratracheal instillation of mycotoxins. Each experimental group comprised six animals. Mean concentrations in pg/mL of cytokines (**A**) TNF-α, (**B**) IL-6 and chemokine (**C**) MIP-1α are presented with bar height. Error bars present 95% confidence interval (CI). Abbreviations: STC—sterigmatocystin, 5-M-STC—5-metoxysterigmatocysin. Control rats were treated with 0.3% DMSO.

The concentrations of pro-inflammatory cytokines TNF-α and IL-6 as well as the chemokine MIP-1α are presented in Figure 3. Single mycotoxins insignificantly decreased TNF-α and IL-6 with respect to the control (*p* > 0.05), while the combination of STC and 5-M-STC returned the concentrations of cytokines to control values. Opposite to cytokines, MIP-1α was more decreased in rats treated with the mycotoxin combination (*p* > 0.05), while in rats instilled with single toxins, the levels of MIP-1α were more similar to control values.

### 2.4. DNA Damage Measured by Alkaline and Neutral Comet Assays

**Figure 4:**
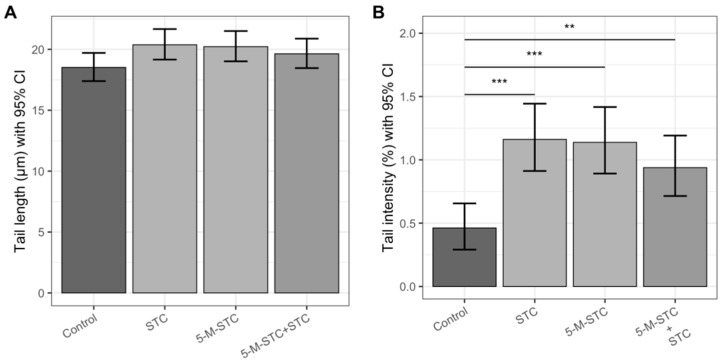
Genotoxic effects obtained by alkaline comet assay in the lungs of Wistar rats following 24 h of intratracheal instillation of mycotoxins. Each experimental group comprised six animals. (**A**) Mean TLs in μm measured in the alkaline comet assay are presented with bar heights, while error bars reflect upper and lower limits of the 95% confidence interval (CI). (**B**) Mean Tis in percentage units measured in the alkaline comet assay are presented with bar heights, while error bars reflect upper and lower limits of the 95% confidence interval (CI). Statistically significant differences between the groups below at the ends of each line are emphasized with asterisks encoding *p* values: *** < 0.001 ≤ ** < 0.01 ≤. Abbreviations: STC—sterigmatocystin, 5-M-STC—5-metoxysterigmatocysin. Control rats were treated with 0.3% DMSO.

**Figure 5:**
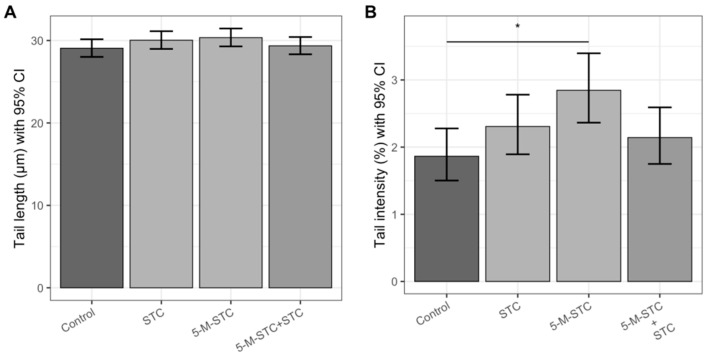
Genotoxic effects obtained by neutral comet assay in the lungs of Wistar rats following 24 h of intratracheal instillation of mycotoxins. Each experimental group comprised six animals. (**A**) Mean TLs in μm measured in the neutral comet assay are presented with bar heights, while error bars reflect upper and lower limits of the 95% confidence interval (CI). (**B**) Mean TIs in percentage units measured in the neutral comet assay are presented with bar heights, while error bars reflect upper and lower limits of the 95% confidence interval (CI). Statistically significant differences between the groups below at the ends of each line are emphasized with asterisks encoding *p* value * <0.05. Abbreviations: STC—sterigmatocystin, 5-M-STC—5-metoxysterigmatocysin.

The results of the genotoxic effects of STC and 5-M-STC measured by alkaline and neutral comet tests are shown as tail length (TL) and tail intensity (TI) in Figure 4 and Figure 5.

Both versions of the comet assay revealed no significant increase in TL (Figure 4A and Figure 5A) in the treatment groups compared to the control. However, the alkaline comet assay showed that the levels of DNA damage, represented by TI, were significantly higher in rats instilled with individual mycotoxins and their combination than in control rats (*p* < 0.001 and *p* < 0.01, respectively). The two-toxin combination provoked lower TI than the individual toxins, but the difference was not statistically significant.

In the neutral comet assay (Figure 5B), a significant increase in TI was observed upon treatment with 5-M-STC alone compared to the control (*p* < 0.05). Although STC and the combination of STC + 5-M-STC caused slightly greater DNA damage compared to the control, the TI increase was not statistically significant. Treatment of animals with STC and the combination of STC + 5-M-STC evoked a lower TI compared to 5-M-STC administered alone, but also did not show a significant difference.

## 3. Discussion

Our recent unpublished results revealed that STC (85%), along with its derivative 5-M-STC (70%), was among the dominant fungal metabolites in the dust of damp dwellings during a post-flood period, linked to the presence of Aspergilli series *Versicolores*, capable of producing both mycotoxins in vitro. That study revealed that the majority of indoor airborne and dustborne isolates of *A. jensenii*, *A. creber*, *A. puulaauensis*, *A. tennesseensis* and *A. venenatus* were capable of producing both STC and its derivative 5-M-STC; the isolates produced two to five times more 5-M-STC than STC. Data on 5-M-STC-producing Aspergilli, as well as the occurrence of this mycotoxin in dust samples of occupational and indoor living areas are scarce. Very few studies reported the occurrence of 5-M-STC in indoor environments in relation to materials contaminated by *A. versicolor* [22,23] but, more recently, the presence of both 5-M-STC and STC in library dust was primarily attributed to *A. jensenii* and *A. creber* [19], which was supported by our results. In library dust, both STC and 5-M-STC were present at similar mean concentrations, ranging from 2.1 to 17.4 µg/kg and 4.8 and 27 µg/kg, respectively [19]. In our study [6], the average concentration of 5-M-STC (215 µg/kg) in indoor dust was eight times higher than the concentration of STC (28 µg/kg), suggesting that Aspergilli could produce significantly larger amounts of 5-M-STC than STC when higher water activity is available in the substrate, which is in line with limited reports in artificially inoculated materials [8,23]. Furthermore, in occupational environments, such as a grain mill, relatively high STC levels (0.06–2.35 μg/g) were detected in dust [7], suggesting that its derivative 5-M-STC might also be present in large amounts. Thus, occupants in specific working environments heavily contaminated with organic dust (e.g., grain storage, grain elevators and mills) may be exposed to high levels of both STC and 5-M-STC, primarily via inhalation.

Having in mind the maximum concentrations of mycotoxins detected in the dust of damp indoor areas in post-flood periods, one single dose per lung weight (l.w.) of STC (0.3 mg/kg l.w.) and 5-M-STC (3.6 mg/kg l.w.), as well as their combination (0.3 + 3.6 mg/kg l.w.), were intratracheally instilled in male Wistar rats. Several toxicity studies in rodents, including mice and rats, available in the literature were done with STC alone [9,12,24], while there are limited results on 5-M-STC in vitro [16,17,25] and no data on its toxicity in vivo. This study shows that naturally occurring concentrations of STC and 5-M-STC alone, as well as their combination, insignificantly increase LDH activity and vascular permeability in rat lungs and we can only speculate that an increase in the dose would produce significant alterations. 

As is well known, TNF-α and IL-6 are pro-inflammatory cytokines produced by lymphocytes, monocytes and epithelial cells [26]. TNF-α is an endogenous pyrogen and immunoregulatory cytokine responsible for the production of interleukins, including IL-6, which leads to the activation of T cells and the differentiation of B cells, as well as immunoglobulin secretion [27,28]. The chemokine MIP-1α plays an important role in the inflammatory process by promoting the recruitment of neutrophils, macrophages and lymphocytes to the site of inflammation [29]. Both TNF-α and IL-6 were insignificantly decreased by STC and 5-M-STC alone in the BALF of rat lungs, and returned to control values following treatment with their combination. In mice, an interperitoneal injection of a 10 times higher dose of STC (3 mg/kg) significantly downregulated the expression of TNF-α and IL-6 in intraperitoneal macrophages, together with a decrease in both cytokines in serum [30]. On the other hand, in mice intratracheally instilled with STC, at half the dose (0.138 mg/kg l.w.) in the present study, it provoked significant inflammation, observed as the infiltration of leukocytes in bronchi, swollen macrophages in alveolar spaces and the upregulated expression of TNF-α and the chemokine MIP-2 [14]. Altogether, this suggests that: (i) STC immunomodulatory effects depend on the applied dose; (ii) in naturally occurring concentrations detected in damp dwellings, STC and 5-M-STC, as structurally similar toxins, may have negative immunomodulatory effects when present alone; (iii) since treatment with STC + 5-M-STC returned pro-inflammatory cytokines and chemokine to control values, an immunomodulatory antagonism within a toxin combination may be expected. Additionally, STC induced an insignificant increase in TNF-α in human THP-1-like macrophages, while co-exposure with β-glucan resulted in a synergistically increased expression of several inflammation-related genes [31], suggesting that mycotoxins may exhibit pro-inflammatory synergistic action with structures of the fungal cell wall that are expected in dust of occupational and indoor living areas. 

Although the STC and 5-M-STC doses used in the present study did not exert significant cytotoxicity and immunomodulation in rat lungs, significant genotoxic action was obtained by two types of comet assay. The principle of the comet assay is based on DNA damage, such as strand breaks, resulting in the extension of DNA loops from lysed nuclei, which form comet-like tails; after alkaline electrophoresis, single-strand breaks dominate, but double-strand breaks are also detected in tails, while neutral electrophoresis indicates the domination of double-strand breaks [32]. Relative TI is the most useful parameter of the comet assay because the tail increases in intensity, but not in length, as DNA damage increases, while TL increases only when tails first become established at a relatively low damage level [33]. Looking at TI, in the alkaline comet assay, both STC and 5-M-STC provoked a similar intensity of DNA damage, although STC was applied at an approximately 10 times lower dose than 5-M-STC, while in the neutral comet assay version, only 5-M-STC evoked significant DNA damage. These results suggest that naturally occurring concentrations of STC alone may induce DNA damage in rat lungs, in which single DNA strand breaks prevail, while 5-M-STC is more responsible for double-strand breaks. In both versions of the comet assay, treatment with the toxin combination resulted in lower TI compared to single mycotoxin treatment, suggesting the antagonistic genotoxic action of STC and 5-M-STC. The present study is the first to report on the genotoxic action of 5-M-STC in vivo, as well as the genotoxic action of the STC + 5-M-STC combination. The nature of DNA strand breaks induced by STC has not been elucidated yet. In porcine primary tracheal epithelial cells treated with STC (50 μM), CYP enzymes produced a reactive STC metabolite [18], which may interact with DNA, leading to DNA single-strand breaks. On the other hand, a recent study revealed that STC at concentrations between 5 and 10 μM possesses unique aggregation properties in water, yielding a strong and specific circular dichroism spectrum [34]. Data showed that STC non-covalently interacts with DNA, most probably by intercalation between base pairs, which may result in DNA double-strand breaks [34]. We can only speculate whether both mechanisms may be in action, depending on the bioavailable concentration of STC and CYP activity. In porcine primary tracheal epithelial cells, 5-M-STC applied at 1 μM was unable to produce CYP-related reactive metabolites [8]. Taking into account the neutral comet assay results obtained in the present study, as well as the structural similarities of 5-M-STC and STC, we may speculate that the intercalation of 5-M-STC yielded a significant amount of DNA double-strand breaks. This hypothesis should be further explored. In human adenocarcinoma A549 cells, both mycotoxins in single treatments at sub-cytotoxic concentrations induced significant reversible and irreversible DNA damage, as measured by comet and micronucleus tests [16]. The mechanism of STC genotoxicity in pulmonary cell lines (A549 and BEAS-2B cells) has been linked to cell cycle arrest in G_2_/S and G_2_/M, in which STC altered the expression of the regulatory protein cyclin and cyclin-dependent kinases [35]. This mechanism of DNA damage might be worth exploring in vivo by applying naturally occurring concentrations of STC and 5-M-STC, as well as their combinations.

In conclusion, STC and 5-M-STC alone and their combination, applied at naturally occurring concentrations detected in damp indoor areas, evoked significant DNA damage, but insignificant cytotoxicity, alterations in vascular permeability and immunomodulation in the lungs of Wistar rats. Changes in the measured parameters after treatment with the STC and 5-M-STC combination suggest their antagonistic interaction. The underlying mechanisms of genotoxicity of single and combined STC and 5-M-STC should be further explored. 

## 4. Materials and Methods 

### 4.1. Experimental Design and BALF Sampling

Adult male Wistar rats (12 weeks old, 300–400 g of body weight (b.w.), mean = 343; mean of lung weight (l.w.) = 1.382 g) were kept in macrolon cages at a controlled room temperature and day/night cycles (22 °C, 12 h, respectively). Before and during the experiment, animals had free access to standard pelleted food (4RF21 from Mucedola, Settimo Milanese, Italy) and tap water. The experiment was approved by the Ethics Committee of the Institute for Medical Research and Occupational Health in accordance with the European Communities Council Directive of 22 September 2010 (2010/63/EU). Animals (N = 24) were randomly divided into four groups (N = 6 in each group) as follows: control (DMSO + PBS); STC; 5-M-STC; and STC + 5-M-STC. Stock solutions of toxins were prepared in DMSO and then diluted to working solutions with PBS. Animals were treated with a single dose of STC (0.4 µg) and 5-M-STC (5 µg), as well as their combination (0.4 + 5 µg) in DMSO/PBS (V = 300 µL), by intratracheal instillation of 300 µL between 8 a.m. and 9 a.m. According to the mean lung weight (l.w.) of rats (1.382 g), doses of toxins were 0.3 mg STC/kg l.w. and 3.6 mg 5-M-STC/kg l.w. Before instillation, animals were lightly anesthetized with isoflurane (Piramal Enterprises LTD, Mumbai, India). Animals were held by hand in the upright position after instillation until awakening, before being placed back in their cages. Animals were sacrificed after 24 h under general anesthesia by Narketan (80 mg/kg body mass (b.m.) and Xylapan (12 mg/kg b.m., i.p.). Lungs were lavaged with ice-cold PBS in 2 × 5 mL and BALF was pulled and centrifuged at 650 G and 4 °C for 10 min. BALF and lungs were frozen at −80 °C until analysis.

### 4.2. LDH Activity Analysis

The measurement of LDH activity in BALF was performed in 96-wellplates using an LDH Assay Kit (Abcam, Cambridge, UK) on a Tecan Infinite M200PRO plate reader (Tecan Austria GmbH, Grodig, Austria). LDH reduces NAD to NADH, which interacts with a specific probe to produce a color. A standard calibration curve was prepared using NADH standard concentrations of 0–12.5 nmol/well. According to the LDH Assay Kit to 50 µL of standard or sample, 50 µL of reaction mix were added, mixed, incubated for three hours and measured in kinetic mode at 450 nm, every 2–3 min for 60 min at 37 °C, protected from light. Absorbance was corrected by subtracting the mean absorbance of the blank from all standards and sample readings. LDH activity was calculated as a concentration of NADH generated by LDH during the reaction time in the volume added to the reaction well.

### 4.3. Albumin Analysis

The measurement of albumin in BALF was performed in 96-well plates using an Abcam Albumin Assay Kit (Abcam, Cambridge, UK) on the Tecan Infinite M200PRO plate reader (Tecan Austria GmbH, Grodig, Austria). The assay is based on the selective interaction between bromcresol green (BCG) and albumin, forming a chromophore that can be detected spectrophotometrically. The signal is directly proportional to the amount present in the serum. A standard calibration curve was prepared using bovine serum albumin (BSA) standard concentrations of 0–75 µg/well. All samples were measured in duplicate. To 50 µL of undiluted serum, 100 µL of diluted BCG were added and plates were incubated at 25 °C for 20 min, protected from light. Absorbance was measured at 620 nm. The mean value of the absorbance of the blank was subtracted from all standards, samples and control readings. The concentration of albumin, in µg/mL, was calculated from the calibration curve of standards.

### 4.4. Cytokines and Chemokine Analysis 

An enzyme-linked immunosorbent assay (ELISA) was employed to measure the levels (pg/mL) of the pro-inflammatory cytokines interleukin-6 (IL-6) and tumor necrosis factor alpha (TNF-α), as well as a chemotactic cytokine, MIP-1α, in BALF. The assays were performed using Rat SimpleStep ELISA^®^ kits for IL-6 (ab234570, Abcam, Cambridge, UK), TNF-α (ab46070, Abcam, Cambridge, UK) and MIP-1α (ab213916, Abcam, Cambridge, UK), following the instructions provided by the manufacturer. To optimize the dilutions of BALF, pilot runs were conducted and a 1:1 dilution was used in all assays. Concentrations of the target proteins IL-6, TNF-α and MIP-1α in the samples were calculated from a standard curve created by plotting the average blank control-subtracted absorbance value for each standard concentration (y-axis) against the target protein concentration (x-axis) of the standard. Concentrations of the standards ranged from 62.5–4000 pg/mL for IL-6, 15.625–500 pg/mL for TNF-α and 7.8–500 pg/mL for MIP-1α. To optimize the dilutions of BALF, pilot runs were conducted and a 1:1 dilution was used in all assays. The samples and standards were processed in duplicate and absorbanceswere measured at a 450 nm wavelength using a microplate reader (PerkinElmer VictorX3, Waltham, MA, USA).

### 4.5. Alkaline and Neutral Comet Assay

Comet assays were performed on the lung samples in accordance with previous protocols [36,37,38]. All of the chemicals used in both comet assays were obtained from Sigma Chemical Company (Sigma-Aldrich, Munich, Germany). Normal melting point (NMP) agarose 0.6% was layered on slides precoated with 1% NMP agarose. Suspensions of lung cells (V = 10 µL per slide) were mixed with 0.5% low-melting point (LMP) agarose, placed on slides and covered with a top layer of 0.5% LMP agarose. After solidification, microgels were immersed in a freshly prepared lysis solution (pH 10.0; 100 mmol/L Na_2_EDTA, 2.5 mol/L NaCl, 1% Na lauroylsarcosinate, 10 mmol/L Tris-HCl, 10% DMSO and 1% Triton X-100) and stored overnight at 4 °C. Upon incubation, in the alkaline version of the comet assay, slides were subjected to 15 min of denaturation (1.5 mol/L NaCl, 1 mmol/L Na_2_EDTA, pH 12.1), followed by 20 min of electrophoresis using the same buffer composition at 0.7 V/cm, 300 mA. In the neutral comet assay, slides were denatured in buffer (300 mmol/L Na-acetate, 100 mmol/L Tris-HCl, pH 8.5) for 1 h at 4–8 °C, followed by 1 h of electrophoresis using the same buffer composition at 4–8 °C, 0.5 V/cm and 10–11 mA. Then, the microgels were neutralized with three changes of 0.4 mol/L Tris-HCl buffer (pH 7.5) at 5 min intervals and stained with ethidium bromide (20 μg/mL). The level of DNA damage in individual cells was assessed with an image analysis system (Comet Assay IVTM, Instem-Perceptive Instruments Ltd., Suffolk, Halstead, UK) using an epifluorescence microscope (Olympus BX50, Tokyo, Japan) under 200× magnification. A total of 100 comets per rat were scored. Tail length (TL) and tail intensity (TI) (i.e., DNA % in tail) were chosen as indicators of DNA damage.

### 4.6. Statistical Analysis

LDH activity, albumin, cytokines, chemokines and comet assay tail length and tail intensity were analyzed using general linear models. The LDH activity model had activity as a dependent variable and group (with levels: control (CTL), sterigmatocystin (STC), 5-metoxysterigamotcystin (5-M-STC) and the combination of sterigamtocystin and 5-metoxysterigmatocystin (STC + 5-M-STC)) as an independent variable. The albumin model had the log_10_-transformed concentration as a dependent variable and group as an independent variable. Cytokines (TNF-α and IL-6) and the chemokine MIP-1α had the log_10_-transformed concentration as a dependent variable, group as a fixed factor and rat identifier as a random factor (to account for dependencies between repeated measures) [39]. For the dependent variable, tail intensity and tail length (for both comet assays) models had log_10_-transformed values. Since some values of tail intensity were equal to zero, 1% was added to all tail intensity values prior to transformation. Within independent variables, group was defined as a fixed factor, and rat identifier and slide identifier (nested within rat identifier) were random factors. 

For all analyzed variables, after fitting models, a *t*-test as a post hoc test was applied on the group factor to assess the differences between each level and control, along with comparisons of the combination of mycotoxins with each mycotoxin [40]. The false discovery rate was controlled with the Bonferroni method. The level of statistical significance was set to 0.05. The results were plotted using ggplot2 package version 3.3.0 (5 March 2020) [41]. All statistical analyses were performed in R software version 3.6.3 (29 February 2020) for statistical computing [42].
